# Definers and drivers of functional high-risk multiple myeloma: insights from genomic, transcriptomic, and immune profiling

**DOI:** 10.3389/fonc.2023.1240966

**Published:** 2023-10-02

**Authors:** Rahul Banerjee, Kara I. Cicero, Sarah S. Lee, Andrew J. Cowan

**Affiliations:** ^1^ Division of Hematology and Oncology, Department of Medicine, University of Washington, Seattle, WA, United States; ^2^ Clinical Research Division, Fred Hutchinson Cancer Center, Seattle, WA, United States; ^3^ Division of Myeloma, Department of Hematology & Hematopoietic Cell Transplantation, City of Hope, CA, United States

**Keywords:** myeloma, induction therapy, cytogenetics, functional, high-risk

## Abstract

Traditional prognostic models for newly diagnosed patients with multiple myeloma (MM), including International Staging System criteria and number of high-risk chromosomal abnormalities, are based on disease characteristics at diagnosis. However, the identification of patients at risk of more rapidly progressive MM is inherently a dynamic assessment. In a subset of patients with MM, adverse disease biology only becomes evident after the failure of first-line therapy. We define this entity as functional high-risk MM (FHRMM), encompassing relapse within 18 months of treatment initiation and/or within 12 months of frontline autologous stem cell transplantation. FHRMM is not adequately captured by traditional prognostic models, and there is a need for better understanding of mechanisms or risk factors for early relapse or progression. In this review, we explore potential definitions of FHRMM before delving into its underlying drivers based on genetic, transcriptomic, and immune cell profiling studies. Emerging data suggest that specific features of both myeloma cells and immune cells can enable the FHRMM phenotype. We conclude our review by discussing ongoing and future studies that seek to identify and intervene upon patients with FHRMM preemptively.

## Introduction

Multiple myeloma (MM), a malignant neoplasm of plasma cells, is marked by considerable heterogeneity in outcomes after diagnosis and initiation of frontline therapy. With Revised International Staging System (R-ISS) criteria, 5-year rates of progression-free survival (PFS) for newly diagnosed patients range from 55% to 36% to 24% depending on staging parameters at diagnosis (1). Similarly, for patients with MM receiving modern induction therapy with four-drug regimens, the presence of high-risk cytogenetics at diagnosis modulates the degree of benefit that patients receive from the addition of novel agents (2–4). The presence of 2 or more high risk chromosomal features at diagnosis appears to confer a particularly negative prognosis, even in the era of quadruplet induction followed by autologous stem cell transplantation (ASCT) (2, 5). Other poor prognostic markers detectable at diagnosis include primary plasma cell leukemia, anaplastic morphology, and soft-tissue extramedullary disease (EMD) (6–8).

**Figure 1:**
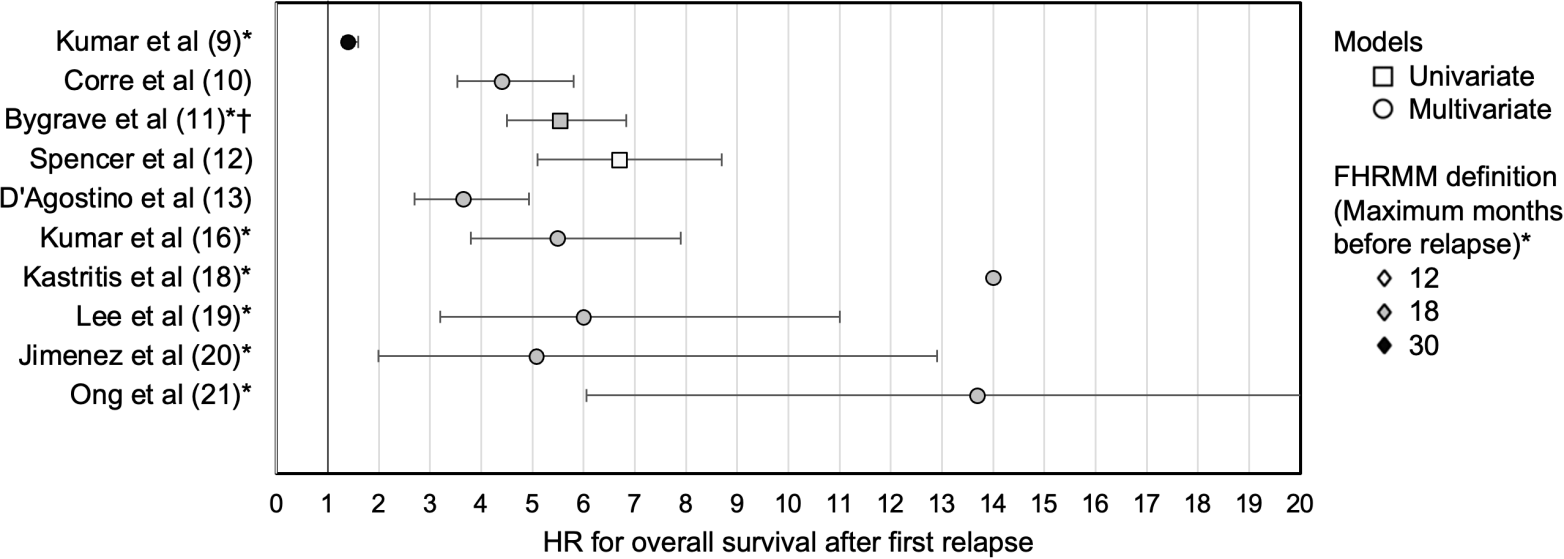
Studies of FHRMM and hazard ratios for overall survival. For studies from **Table 1**, HR with 95% CIs are depicted for overall survival following first relapse for patients with FHRMM versus patients with later relapses. Studies where HR and 95% CI were not reported are not shown. * 6 months added to PD criteria for FHRMM if defined from ASCT rather than start of therapy. † RR reported. ASCT, autologous stem cell transplant; CI, confidence interval; FHRMM, functional high-risk multiple myeloma; HR, hazard ratio; PD, progressive disease; RR, relative risk.

In contrast, functional high-risk multiple myeloma (FHRMM) is only defined through dynamic assessment of disease kinetics after treatment initiation. At its broadest level, FHRMM could potentially include both patients with primary refractory MM as well as patients with early relapse following first-line therapy. In this Review, we focus on the second definition of FHRMM with an emphasis on early relapse. Although the definition of early relapse varies between studies as shown in **Table 1** (9–21), we define FHRMM as progressive disease (PD) within 18 months of treatment initiation and/or PD within 12 months of frontline ASCT (10, 11, 13, 16–21). As illustrated in **Figure 1**, these studies have uniformly shown that FHRMM status following frontline therapy is associated with worsened overall survival (OS) thereafter (9–21).

**Table 1:** Selected studies of FHRMM and overall survival.

Study	*n*	PD criteria for FHRMM	% FHR MM	OS, months (FHRMM)	OS, months (non-FHRMM)
**Kumar** **et al** (9)	256	<24 months of ASCT	37%	45 (43-48)	114 (108-122)
**Corre** **et al** (10)	2627	≤18 months of starting therapy	19%	45*	>52*
**Bygrave** **et al** (11)	1349	≤12 months of ASCT	13%	26 (21-28)	91 (85-NR)
**Spencer** **et al** (12)	1320	≤12 months of starting therapy	9%	20 (15-25)	61 (58-NR)
**D’Agostino** **et al** (13)	926	≤18 months of starting therapy	21%	33	NR (NR-NR)
**Helm** **et al** (14)	575	≤18 months of ASCT	29%	35 (28-45)	127 (95-NR)
**Majithia** **et al** (15)	511	≤12 months of starting therapy	16%	21 (16-27)	NR (96-NR)
**Kumar** **et al** (16)	494	≤12 months of ASCT	24%	27 (23-31)	91 (71-111)
**Wu** **et al** (17)	423	≤12 months of ASCT	31%	15	40
**Kastritis** **et al** (18)	297	≤12 months of ASCT	15%	18	175*
**Lee** **et al** (19)	257	≤12 months of ASCT	14%	18	NR
**Jimenez-Zepeda** **et al** (20)	184	≤12 months of ASCT	36%	20 (16-25)	93 (75-111)
**Ong** **et al** (21)	163	≤12 months of ASCT	16%	22	NR

For each study, median overall survival is listed; where reported by study authors, 95% confidence intervals are provided as well. *Based on visual analysis of Kaplan-Meier plots.

ASCT, autologous stem cell transplantation; FHRMM, functional high-risk multiple myeloma; NR, not reached; OS, overall survival; PD, progressive disease.

Given these findings, multiple groups have attempted to identify clinical risk factors that can predict FHRMM status following frontline therapy (22–31). Potential risk factors from these studies include R-ISS staging (or specific components thereof), performance status, specific myeloma-defining events, suboptimal induction regimens, or suboptimal depths of response. However, these scoring systems vary widely in terms of FHRMM definitions, relevant covariates, and analytic performance. One such calculator from the GIMEMA group, the Simplified Score to Predict Early Relapse in Multiple Myeloma (S-ERMM), has shown conflicting results in different datasets (23, 27, 29). As such, better tools to define patients at high risk of early relapse are needed. In this Review, we explore emerging evidence from genomic, transcriptomic, and immune cell profiling studies to identify these patients. Pre-clinical and translational studies suggest that both myeloma cells and immune cells play large roles in enabling early relapse. Our review of the multi-omic landscape of FHRMM highlights the need for both more precise prognostic models to capture the full spectrum of risk in MM as well as future studies that intervene on FHRMM biology before clinical relapse occurs.

## Myeloma cell biology in functional high-risk myeloma

Gene expression profiling (GEP) assays, which look at the expression of targeted genes by levels of messenger RNA (mRNA) expression within MM cells, may be able to predict FHRMM. Two such GEP tools, the GEP70/UAMS70 assay (marketed as MyPRS) and the EMC92 assay (marketed as SKY92), are commercially available in the United States (US). In one study of 94 patients using the MyPRS assay, rates of relapse within 12 months of induction were 28% in patients with high-risk MyPRS scores versus only 2% in patients with low-risk MyPRS scores (32). Interestingly, early relapse occurred in 30% of patients with high-risk MyPRS scores and low-risk conventional cytogenetics; in contrast, no patients with low-risk MyPRS scores and high-risk cytogenetics had early relapse. In a secondary analysis of the Myeloma XI trial, early relapse occurred in over a third of patients with high-risk SKY92 scores regardless of whether they received post-transplant lenalidomide or not (33). Another 17-gene panel, the REL-17 signature, has been validated to predict patients with >60% probability of relapsing within 12 months of ASCT (17). However, it is worth noting that the routine use of these tests in the US has historically been limited by issues around insurance reimbursement (34, 35).

In addition to these multi-gene panels, specific genes associated with cell growth or tumor suppression are often implicated in the FHRMM phenotype. Several groups have sought to identify culpable genes or gene families using GEP as well as more comprehensive genomic sequencing. For example, a recent analysis of genomic data from the Multiple Myeloma Research Foundation CoMMpass dataset demonstrated that genes involved in the IL-6/JAK/STAT3 signaling pathway are associated with FHRMM (36). Genes associated with glycolysis, hypoxia tolerance, and oxidative stress are enriched in FHRMM as well (36–38). Unsurprisingly, DNA damage repair pathways including *TP53* (whether mutated or lost) are disrupted in patients with FHRMM at higher proportions as compared to patients with relapse occurring at later timeframes (13, 37, 38). In one study comparing FHRMM patients at first relapse versus heavily pre-treated non-FHRMM patients (with a median of 5 prior lines of therapy), biallelic inactivation of *TP53* or *RB1* was more common (44% versus 30%) in the FHRMM subgroup than the heavily pre-treated subgroup (38).

Beyond genes focused on cell growth or tumor suppression, a few miscellaneous genes additionally warrant discussion. Mutations in genes involved with epithelial-mesenchymal transitions may predispose patients to early relapse: for example, integrin-ɑ8 has been found to be enriched in FHRMM and drives MM cell proliferation and invasion (38, 39). The noncoding RNA transcript *MALAT1*, which may similarly be involved in this process, is found at higher levels among patients with early relapse (40). Other noncoding mRNA transcripts, for example miR-181a which is found on chromosome 1q, have also been implicated in this setting (41). High levels of miR-193a-5p circulating in peripheral blood are conversely associated with lower rates of early relapse, although this may be a function of this particular transcript’s association with proteasome inhibitor sensitivity (42). Some of these findings may have relevance to conventional markers of disease aggressiveness, for example mesenchymal transition as a driver of EMD. However, further research is needed to validate these associations.

## Immune cell biology in functional high-risk myeloma

The tumor immune microenvironment (TME) plays a key role in MM pathogenesis and is also important in mediating the risk of relapse after effective therapy (43). Recent analyses have demonstrated that, with time and exposure to different MM-directed therapies, the TME evolves just as MM cells themselves evolve. Specifically, in a study of 39 patients including newly diagnosed patients and triple-class-refractory patients, TME analyses using mass cytometry, cytokines, and RNA sequencing of bone marrow samples demonstrate that the TME becomes less functional over time: in particular, increasing numbers of senescent T cells and fewer early-memory T cells are detected during later lines of therapy (44). As detailed below, several studies have examined immune cell subsets in similar detail with an emphasis on early relapse after induction therapy or after ASCT. These types of analyses can help us better understand TME-based drivers of FHRMM that are not reliably captured by traditional prognostic models.

Most research to date with regard to immune cell profiling in MM has focused on T-cell types and phenotypes. For example, in one study of bone marrow aspirates from newly diagnosed patients with MM, PFS was almost three times worse in the subset of patients with regulatory T-cell (T_reg_) frequencies above the median (45). These patients also had higher levels of PD-1 and LAG-3 expression (markers of T-cell exhaustion) as well. Specific to FHRMM, an analysis of marrow-infiltrating T-cells at Day +100 after ASCT identified a distinct subgroup with shorter PFS; this subgroup had increased T_reg_ cells and greater levels of regulatory markers such as ICOS, PD-1, and LAG-3 on effector cells (46). Moving beyond T_reg_ cells, a xenograft model of ASCT has found that inhibitory receptor expression on CD8+ T cells as well as downregulation of the costimulatory receptor CD226 (DNAM-1) were also associated with MM progression (47). In this study, these DNAM-1 negative CD8+ T cells had an exhausted phenotype with increased TIGIT and PD-1 expression. In an analysis of post-ASCT lymphocyte composition and function involving 55 patients, patients who ultimately relapsed after ASCT had greater numbers of exhausted CD8+ T cells (with upregulation of CD57 and PD-1) detectable even prior to clinical detection of PD (48). One study of 58 MM patients identified distinct TME patterns after ASCT via cytometry with time of flight (CyTOF) analyses and found that those with increased levels of naïve and terminally differentiated T cells had comparatively worse outcomes regardless of the presence of high-risk chromosomal abnormalities (49).

The contribution of B cells to the FHRMM phenotype is less well defined. While regulatory B-cells (B_reg_) are found at higher concentrations within the bone marrow of newly diagnosed MM patients as compared to patients in remission or healthy volunteers, B_reg_ cells are typically eradicated by therapy and remain undetectable at relapse (50, 51). Similarly, B-cell subset analyses did not play a significant role in identifying unique TME clusters that predict good versus poor responses to therapy (44). This general lack of impact may be related to global B-cell suppression and dysfunction that occurs early in MM pathogenesis in most cases, even for patients without clear immunoparesis (44, 52). Alternatively, because low B_reg_ frequencies are associated with worsened OS but not worsened PFS in elderly patients with MM, B cell subset analyses may be more pertinent to other competing disease processes (e.g., infectious complications) than they are to the kinetics of MM progression (53, 54).

In contrast to B cells, natural killer (NK) lymphocytes clearly play important roles in the immune response against MM (55–57). As such, it is unsurprising that the dynamics of NK cell reconstitution following transplantation has been associated with outcomes in MM. In a study of 114 patients with MM, patients with low NK cell counts in peripheral blood (<100 cells per microliter) 1 month post-ASCT had worsened PFS compared to patients with NK cell counts of 100-200 per microliter (58). Similarly, an analysis of adaptive NK cells (long-lived NK cells with properties of immunologic memory against viruses such as cytomegalovirus) showed that greater absolute numbers of adaptive NKs was associated with a lower risk of relapse following ASCT (59). In brief, lower numbers of NK cells during post-ASCT immune reconstitution may be associated with greater risk of relapse. Conversely, higher levels of NK cells three months after ASCT is associated with higher rates of measurable residual disease (MRD) negativity (60). In addition to quantitative NK cell frequencies, NK cell phenotypes may also play a role as well: in one study, patients with higher levels of terminally differentiated NK cells after ASCT had worsened PFS than those with more immature NK cells (56).

Non-lymphoid immune cells may also enable or protect against FHRMM in certain cases, although conclusions are difficult to draw given the complex web of myeloid-lineage cellular interactions in the tumor microenvironment. For example, in a detailed analysis of patient marrow samples following quadruplet induction, MRD negativity was associated with higher levels of monocytes as well as plasmacytoid dendritic cells within the bone marrow (61). In contrast, high levels of circulating monocytes have been a poor prognostic feature in several studies (62, 63). The types of monocytes within the blood and marrow may matter as much as their quantities, given that non-classical monocytes (CD16+CD14^dim^) may drive myeloma cell growth and increased tumor burden (64, 65). In contrast, classical monocytes (CD14+CD16-) may have the opposite effect (66, 67). Differentiated monocytes show a similar range of behaviors depending on their phenotype, as evidenced by the presence of M2-polarized tumor-associated macrophages (associated with immunosuppression) as a marker of earlier relapses (68, 69). However, even pro-inflammatory M1-polarized macrophages may lead to downstream promotion of stem-like MM progenitor cells and bortezomib resistance through complex cytokine signaling pathways (70). Evidently, more studies of monocyte-lineage cells and their overlapping impacts on the FHRMM phenotype are required.

Finally, serum cytokine levels may also play a role in risk stratification for FHRMM. In an analysis of 188 Chinese patients with newly diagnosed MM, baseline interleukin (IL)-10 levels over 169.96 pg/ml were associated with inferior PFS and OS (71). In a retrospective study, serum IL-2, IL-4, IL-6, IL-10, IL-17a, TNF-alpha, and IFN-gamma levels were markedly elevated in newly diagnosed MM patients compared to healthy controls; in a multivariate analysis, IL-6 and IL-17a were prognostic factors for survival (72). Soluble IL-2 receptor (sIL-2r), which normally stimulates lymphocyte proliferation after binding IL-2, is generally released from lymphocytes via cleavage of the alpha chain (CD25) and is increased in several malignancies (73). sIL-2r competes for binding of IL-2 with the IL-2 receptor and thus may play a role in blocking anti-tumor immune based activities. In an analysis of 88 patients with newly diagnosed MM, sIL-2r levels were an independent risk factor for PFS (74).

Taken together, several studies have shown an important role for immune cells within the TME with respect to treatment failure and progression. However, these TME characteristics are not reliably captured by our current risk-stratification systems for MM. Most studies have focused on T_reg_ frequencies or markers of T-cell exhaustion such as PD-1 or TIGIT, but NK cell properties in the post-ASCT setting impact the risk of early relapse as well. Finally, levels of specific serum cytokines, initially altered in newly diagnosed MM compared to healthy controls, may also be prognostic for survival. The interplay between adverse features of immune cells and myeloma cells remains unclear: is T-cell exhaustion an independent phenomenon in patients with FHRMM, or is it being driven by biological features of MM cells that remain incompletely characterized? Regardless, incorporating these TME factors as novel biomarkers for early progression may help better refine prognostic models and address the unmet need around FHRMM identification.

## Mitigating the risk of functional high-risk myeloma

Strategies to identify a distinct FHRMM phenotype through multi-omic analyses of both myeloma cells and immune cells are only a first step; ideally, patients should also be managed differently once FHRMM is recognized clinically. Such FHRMM-specific management should emphasize not just more aggressive therapy, but rather more personalized therapy tailored to FHRMM’s unique disease and immune biology as summarized in this review. In **Table 2**, we outline four potential strategies to intervene on these patient populations both before and after their diagnosis of FHRMM, spanning from improved patient counseling to enhanced surveillance to altered therapeutic strategies. Given the paucity of published evidence surrounding the drivers of FHRMM, robust trials are still needed before these therapeutic strategies can be widely adopted.

**Table 2:** Potential strategies to intervene on FHRMM.

Strategy	Theoretical rationales
Better identification of patients with an FHRMM phenotype* at diagnosis	* Patients can receive more accurate counseling at diagnosis regarding expected durability of responses * Strategies used in cytogenetic high-risk MM, for example doublet maintenance, can be considered
Enhanced surveillance for early relapse in patients with an FHRMM phenotype*	* Early identification of relapse may theoretically lower tumor burden present at later timepoints * Clinical relapse, e.g. with bone pain or fractures, may theoretically be avoidable if relapse diagnosed early
**More aggressive MM-directed therapy once FHRMM evident**	* Historical data suggest that, for patients with FHRMM, subsequent overall survival is low (9–21) * These patients can potentially be triaged toward novel investigational agents as noted below
**Precision therapies targeted at underlying drivers of FHRMM (not yet proven)**	* Targeting therapy for patients with genetic mutations through the MyDrug study (75) * For example: inhibition of the IL-6/JAK/STAT3 pathway overexpressed on MM cells in FHRMM * For example: PD-1 or TIGIT inhibition given their overexpression on exhausted T cells in FHRMM

*The FHRMM phenotype is defined as patients with an a priori higher risk of early relapse based on the emerging biomarkers and assays described in this review.

FHRMM, functional high-risk MM; MM, multiple myeloma.

**Figure 2:**
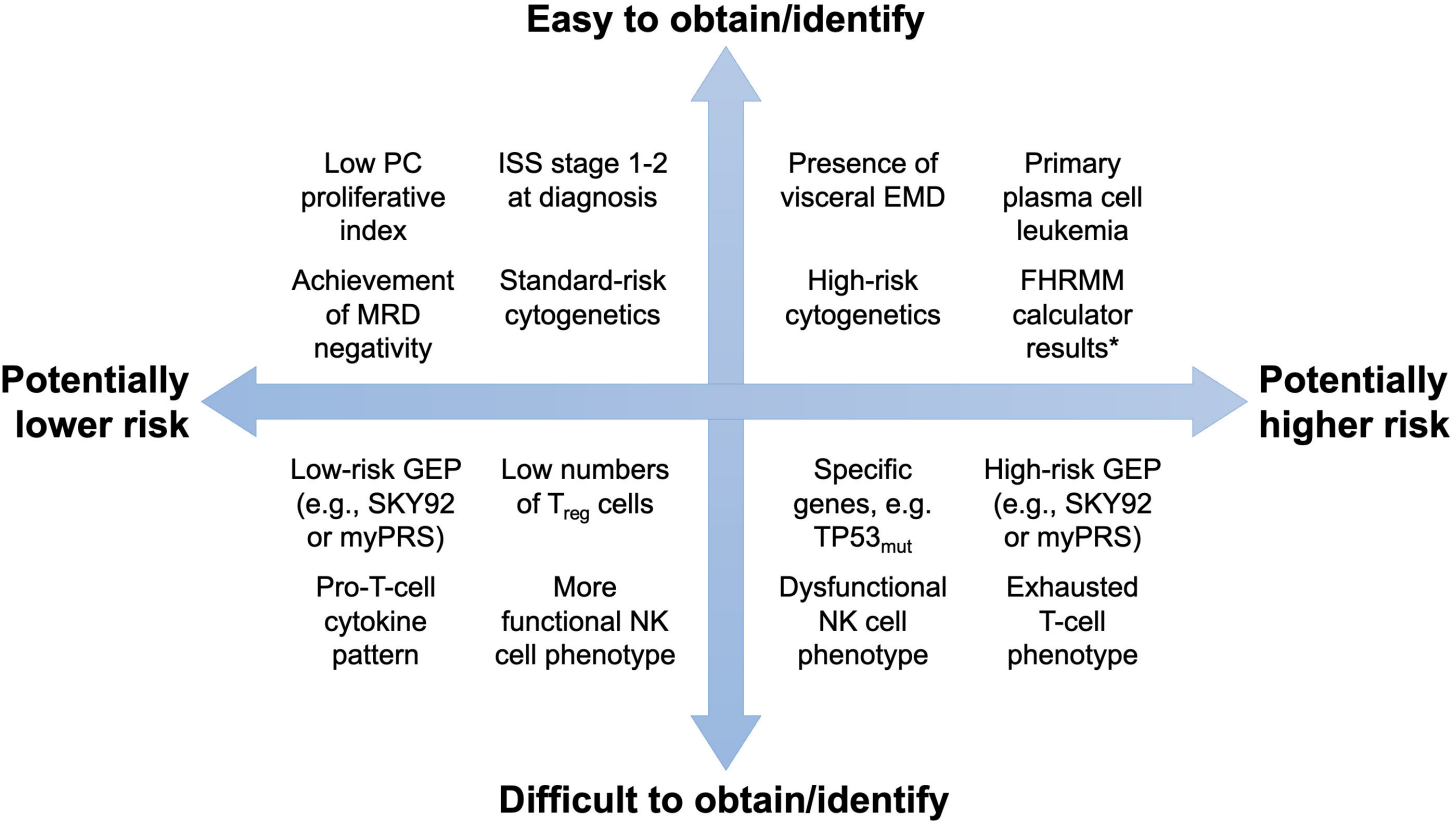
Potential tools to predict the FHRMM phenotype. Tests and tools are organized by their ability to predict the FHRMM phenotype (x-axis) as well as their ability to be obtained or identified in routine clinical practice (y-axis). * For example, the S-ERMM, EBMT, and CIBMTR calculators summarized in this review. EMD, extramedullary disease; FHRMM, functional high-risk multiple myeloma; GEP, gene expression profiling; ISS, International Staging System; MRD, measurable residual disease; NK, natural killer; PC, plasma cell; TP53_mut_, mutated TP53 gene.

Firstly, for patients at higher risk of FHRMM based on the factors identified in **Figure 2**, can we modify their treatment approach *a priori* during frontline therapy? Many trials have already employed multi-drug maintenance or consolidation therapy for patients with cytogenetically defined high-risk disease at diagnosis (76, 77). More recently, the MUKnine OPTIMUM trial included a high-risk SKY92 GEP profile alongside ultra-high-risk cytogenetics or plasma cell leukemia as eligibility criteria for an intensive induction and extended consolidation regimen (78). With regard to FHRMM, retrospective studies have identified the use of multi-drug maintenance or post-ASCT consolidation as protective factors against early relapse (13, 18). Given that GEP assays are not always available or reimbursable, at least three risk calculators (S-ERMM, CIBMTR, and EBMT) based on easily obtainable baseline parameters may be able to guide FHRMM-oriented decision making in future trials (22–31). Conversely, to the extent that patients who achieve deep remissions or MRD negativity during first-line therapy are less likely to experience rapid relapses thereafter, predictive calculators to identify these patients may also identify patients who will be at low risk of FHRMM (79, 80). As of now, we suggest that these calculators be used to guide further prospective studies before they are routinely deployed in clinical care to guide the escalation or de-escalation of frontline treatment.

Secondly, for patients who are suspected of having an FHRMM phenotype, should we screen these patients more aggressively for relapse during the maintenance phase of therapy? Enhanced surveillance with more frequent cross-sectional imaging or bone marrow biopsies may theoretically lead to relapse detection earlier, although the clinical benefit of this has not yet been established. Emerging serum biomarkers may be able to predict PD months before it occurs clinically, for example rising levels of proteins such as DKK1 and sclerostin involved in pathologic bone remodeling (81). Increasing levels of soluble B-cell maturation antigen, a known biomarker of plasma cell burden, may be able to identify impending relapse as well (82). Finally, several groups have investigated the implications of increasing levels of MRD burden on sequential bone marrow biopsies as a harbinger of relapse (83–85). For patients suspected to have an FHRMM phenotype and a correspondingly higher risk of relapse in any given time period, longitudinal assessments of these emerging biomarkers may be justified and even cost-effective.

Thirdly, should we change our approach to next-line therapies once early relapse has occurred? Several studies have found that subsequent PFS and OS are poor in FHRMM (9–21). On a simple level, more drugs in combination are superior when it comes to partially mitigating this risk of poor outcomes in FHRMM. For example, secondary analyses of the randomized ASPIRE and IKEMA trials demonstrate that triplets outperform doublets with regard to PFS among patients with FHRMM at study enrollment (86, 87). In this vein, the ongoing multi-arm MyDrug study conducted by the Multiple Myeloma Research Foundation (clinicaltrials.gov ID: NCT03732703) utilizes mutational analysis with targeted therapies in multi-drug regimens specifically for patients with FHRMM (75). Similarly, the Australasian Myeloma Research Consortium is running the IBIS trial of isatuximab and dexamethasone alongside the cereblon E3 ligase modulator (CELMoD) ibderdomide in patients with FHRMM as well (88). We eagerly await the results of these and other investigations in this space.

Alternatively, given the contribution of immune cell dysfunction within the TME as described above, adoptive cellular immunotherapy may play a particularly important role in managing FHRMM. Idecabtagene vicleucel and ciltacabtagene autoleucel, two chimeric antigen receptor T-cell (CAR-T) therapies currently approved in the US for patients who have received 4+ prior lines of therapy, have been studied as second-line therapy in FHRMM through the KarMMa-2A and CARTITUDE-2B trials, respectively (89, 90). In both single-arm cohorts, these CAR-T therapies behaved comparably to the corresponding KarMMa and CARTITUDE-1 trials involving more heavily pre-treated patients (91, 92). This suggests that CAR-T therapy may be a valuable option for selected patients with FHRMM as second-line therapy. Even allogeneic stem cell transplantation, a modality rarely used in the modern era, may have a role in selected patients with FHRMM (93).

Finally, can precision medicine be used some day to target specific pathways implicated in FHRMM? For example, the aforementioned JAK/STAT3 pathway has been shown to be activated preferentially in cells from patients with FHRMM (36, 37). Ruxolitinib, a known inhibitor of the JAK/STAT3 pathway approved for myelofibrosis, has been shown in a Phase 1 trial to lead to responses in approximately a third of patients with relapsed/refractory MM (94). Interestingly, preclinical studies suggest that ruxolitinib may also lower PD-L1 expression on MM cells and interfere with other pro-growth pathways (95, 96). For patients with FHRMM in the setting of mutations in genes such as RAF or the CDK family, the ongoing MyDrug study is investigating the addition of targeted therapies in addition to anti-MM regimens (75). In patients with slow NK cell reconstitution after ASCT, autologous NK cell infusions (after *ex vivo* expansion) or NK-specific cytokine agonists may be therapeutic options as well (97, 98). With regard to reducing T cell dysfunction, pre-clinical models suggest that TIGIT inhibition on T cells may work to prevent relapse (47, 99, 100). At least one clinical trial (clinicaltrials.gov ID NCT05289492) of TIGIT inhibition in relapsed MM is under way (101). As a caveat, checkpoint inhibition in MM has a mixed history given the premature closure of the KEYNOTE-183 and KEYNOTE-185 trials for excess risk (102, 103). As such, future strategies of checkpoint inhibitors in FHRMM should ideally be paired with advanced diagnostic tools to better select patients who are more likely to benefit from these targeted approaches.

## Discussion

In clinical practice, one of the most vexing characteristics of FHRMM is its unexpectedness. For patients with known risk factors such as high-risk cytogenetics, treatment is often tailored to their aggressive phenotype in terms of consideration of strategies such as tandem ASCT or dual PI-IMID maintenance. In contrast, FHRMM often occurs in patients without such risk factors. As shown in **Table 3**, approximately 20-40% of patients with FHRMM have ISS stage 1 disease (10, 11, 13, 14, 18, 19). Similarly, in several studies, over half of patients with FHRMM have standard-risk cytogenetics at diagnosis (10, 13, 18, 19, 104). These are patients for whom several years of remission after frontline therapy might have been expected, and early relapse is devastating in its own right but moreover because FHRMM confers a poor prognosis thereafter. Better identification of these patients *a priori* is an important first step toward trials of preemptive treatment intensification, novel therapeutic agents, or precision oncology approaches as outlined in this review.

**Table 3:** Overlap between FHRMM and traditional prognostic markers.

Study	FHRMM *n*	% ISS 1	% ISS 2	% SR FISH*	% LDH WNL
**Kansagra** **et al** (104)	1260	N/A	N/A	73%^a^	N/A
**Corre** **et al** (10)	496	22%	45%	67%^b^	N/A
**D’Agostino** **et al** (13)	191	24%	33%	74%	91%
**Bygrave** **et al** (11)	174	22%	42%	28%	N/A
**Helm** **et al** (14)	154	32%	40%	N/A	68%
**Majithia** **et al** (15)	82	58% (combined)		34%	65%
**Kastritis** **et al** (18)	43	31%	33%	68%	74%
**Lee** **et al** (19)	35	24%	53%	52%	N/A
**Wei** **et al** (26)	30	37% (combined)		N/A	57%
**Panopoulou** **et al** (105)	21	N/A	N/A	33%^c^	N/A

*High-risk FISH defined as del(17p), t(4;14), t(14;16), except in the following studies:

a del(17p), t(4;14), t(14;16), t(14;20), gain(1q), hypoploid.

b del(17p), t(4;14).

c del(17p), t(4;14), t(14;16), t(14;20), gain(1q).

FHRMM, functional high-risk multiple myeloma; FISH, fluorescent in situ hybridization; ISS, International Staging System; LDH, serum lactate dehydrogenase; N/A, not available; SR, standard risk; WNL, within normal limits (as calculated by authors of the presented study).

Considering all the factors reviewed thus far, two overarching conclusions can be drawn about FHRMM and risk assessment in MM. First, assessing disease risk is a dynamic process that current prognostic models do not fully capture. Most of our prognostic models in plasma cell disorders, both in active MM and in precursor conditions such as smoldering myeloma, focus on disease parameters at diagnosis and not their kinetics over time. FHRMM is by definition a longitudinal diagnosis, and further studies of patients with FHRMM are required to create dynamic risk assessment systems accordingly. Second, given that we often change our treatment approaches for patients with traditionally defined high-risk MM, FHRMM arguably represents a shortcoming of our current risk models. As noted in **Table 3**, over half of patients who ultimately develop FHRMM are misclassified as having standard-risk disease biology at diagnosis based on the absence of selected cytogenetic abnormalities. Incorporation of novel biomarkers outlined into traditional staging models may help better stratify these patients to avoid undertreatment of patients at high risk of developing FHRMM.

Importantly, we intentionally defined FHRMM narrowly by focusing on early relapse after induction therapy and/or ASCT. However, if characterized more broadly to include any upstaging of risk based on disease kinetics over time, FHRMM could also include patients with primary refractory MM as well as patients with insufficient responses to induction therapy. Patients with primary refractory MM have poor outcomes even in the modern era of CD38-targeted monoclonal antibody incorporation into treatment regimens (106–109). Encouragingly, use of ASCT in the second line for these patients despite frontline refractoriness has recently been associated with improved outcomes (110). Failure to achieve at least a partial response with induction therapy has similarly been associated with worsened outcomes in several studies, although data do not generally support treatment intensification outside of planned ASCT when this occurs (111, 112). Whether the underlying disease and TME biology of these scenarios is similar to the pathophysiology of early relapse discussed in this review is unknown.

A key limitation of our review is its focus on genomic, transcriptomic, and immune drivers of FHRMM. Other diagnostic tools may play a key role in identifying FHRMM as well. For example, the plasma cell proliferative index, while not technically a genomic test *per se*, has been shown to predict early relapse among patients who do not achieve a complete response after ASCT (113). However, this test is not routinely available outside of select US institutions. Given the increasing sensitivity of peripheral blood flow cytometry assays, multiple groups have also demonstrated that circulating plasma cells are a predictor of early relapse whether detected at diagnosis or during stem cell collection (37, 114–116). Advanced imaging modalities such as whole-body diffusion-weighted magnetic resonance imaging may play a role in FHRMM prognostication as well, given that persistence of focal lesions after induction may be indicative of a worsened prognosis (117, 118). Finally, organoid-based models and *ex vivo* assays to assess drug sensitivity may also be helpful in determining the causes of treatment failure in earlier lines of therapy (119–122) Further research is needed to validate these tools and increase their availability for MM physicians across the globe.

In conclusion, FHRMM is a dynamically defined state of high risk defined by multiple factors. FHRMM is unequivocally associated with worsened OS after its emergence, but identification of these patients *a priori* remains a challenge with conventional risk prognostication systems. Both myeloma cell factors and immune cell factors have been implicated in the pathophysiology of early relapse. Larger multi-omic prospective data sets in newly diagnosed MM and early relapsed MM, building on some of the data presented in this review, will help refine prognostic models for these patients in coming years. Thankfully, studies of both diagnostic strategies to identify FHRMM as well as therapeutic strategies to mitigate these patients’ subsequently poor prognoses are under way.

## Author contributions

RB and AC conceptualized the research idea. All authors contributed to the article and approved the submitted version.
